# Research on the Eco-Efficiency of Rice Production and Its Improvement Path: A Case Study from China

**DOI:** 10.3390/ijerph19148645

**Published:** 2022-07-15

**Authors:** Malan Huang, Linlin Zeng, Chujie Liu, Xiaoyun Li, Hongling Wang

**Affiliations:** 1 School of Business, Hubei University, Wuhan 430062, China; wanghongling1999@163.com; 2 China Agricultural Carbon Emission Reduction and Carbon Trading Research Center, Hubei University, Wuhan 430062, China; 3 College of Economics and Management, Huazhong Agricultural University, Wuhan 430070, China; zengll@webmail.hzau.edu.cn (L.Z.); liuchujie@webmail.hzau.edu.cn (C.L.); lixiaoyun@mail.hzau.edu.cn (X.L.)

**Keywords:** rice, eco-efficiency, life cycle assessment (LCA), sensitivity analysis, green production, China

## Abstract

The eco-efficiency of rice production is an important indicator in the measurement of sustainable rice development. Scientific evaluation of the eco-efficiency of rice production facilitates accurate evaluation of the real level of rice ecosystems to realize efficient utilization of agricultural resources. This paper measured the eco-efficiency of farms growing rice using both the life cycle assessment (LCA) and the data envelopment analysis (DEA) methods based on survey data from 370 farms mainly growing rice conducted in 2020 in the Hubei Province, the middle reaches of the Yangtze River in China. Then, sensitivity analysis and scenario analysis were carried out on the comprehensive index of the rice environmental impact and eco-efficiency of rice production, respectively. The results indicate that the comprehensive index of the rice environmental impact was 2.0971. Water toxicity, soil toxicity and eutrophication were the main influencing factors. The mean value of the eco-efficiency reached 0.51. More specifically, the proportion of farms in the low-, middle- and high-efficiency groups was 87.03%, 1.89% and 11.08%, respectively, with mean values up to 0.42, 0.86 and 1.14, respectively. A sensitivity analysis revealed that the pesticide sensitivity was higher than the fertilizer sensitivity in terms of the environmental impact sensitivity of rice systems. When comprehensively considering environmental and economic benefits, the fertilizer sensitivity was higher than that of pesticides. Moreover, reducing the application of both fertilizers and pesticides by 50% could promote the eco-efficiency of rice production systems by 6%, and the value could reach 0.54. Thus, reducing the application of fertilizers and pesticides and improving the utilization efficiency are effective ways to improve green rice production.

## 1. Introduction

China is the largest rice producer and consumer worldwide with sown areas accounting for approximately 19% of the global sown area, and more than 65% of the Chinese population consuming rice as a staple food. The total rice production in 2020 reached 211.86 million tons, accounting for 31.64% of the total grain production [1]. For a long time, the use of agrochemicals, especially fertilizers, has played a historic role in increasing rice yields. However, poor management of agrochemicals, especially excessive application of fertilizers and pesticides, has caused problems such as the deterioration of rice quality, unstable yields and environmental degradation [2]. To promote the sustainable transformation of agricultural operation modes, the Chinese Ministry of Agriculture and Rural Affairs has issued zero-growth policies targeting fertilizers and pesticides since 2015. Five years later and by the end of 2020, China has successfully achieved the desired goal of reducing the commercial use of fertilizers and pesticides and significantly increasing its utilization efficiency. Thus, the effect of promoting the high-quality development of the planting industry is obvious. By scientific calculation, in 2020, the fertilizer utilization efficiency for the main grain crops in China reached 40.2%, 5% higher than that in 2015, and the utilization efficiency of pesticides was 40.6%, 4% higher than that in 2015. However, there remains a large gap in the developed countries, such as European countries and the United States [3]. The report of the 19th National Congress pointed out that ecological civilization construction will enable sustainable development of the Chinese nation, and the philosophy whereby clear waters and green mountains are as good as mountains of gold and silver must be established and practiced [4]. Under the severe conditions of the rural ecological environment, agricultural economic development should maintain overall coordination among the agricultural input and output, resource consumption and environmental protection [5].

The environmental problems attributed to rice production have attracted widespread attention [6]. It is a major challenge to identify a development path to achieve a sustainable increase in rice production while reducing environmental costs. A low production efficiency and excessive use of agrochemicals are the main causes of environmental pollution in rice production. Previous studies have investigated the ecological and environmental impacts of heavy inputs of chemicals during the rice production process [7], including climate warming, environmental acidification, eutrophication and heavy metal pollution [8,9,10], in addition to other ecological and environmental problems caused by unreasonable straw return [11] and irrigation patterns [12]. Currently, life cycle assessment (LCA) and data envelopment analysis (DEA) methods are typically combined to measure the eco-efficiency of specific agricultural products, such as soybeans [13], wheat [14], cotton [15], rapeseed and sunflower [16]. However, in terms of rice production efficiency, existing research has focused on the traditional production efficiency [17,18], and only a few studies have considered the eco-efficiency of rice production [19,20]. In this study, rice eco-efficiency is defined as the relationship between inputs, economic outputs and environmental undesired outputs of rice production in the rice production process.

Many scholars have achieved abundant research results on the agricultural eco-efficiency [21,22], and the corresponding evaluation methods are becoming increasingly scientific and reasonable, which is a meaningful contribution to the field and yields great reference value. However, there remains room for improvement in the existing research. First, in terms of the research scale, due to the limitation of data acquisition, previous research on the agricultural eco-efficiency in China has mainly focused on the macro national and provincial agriculture eco-efficiencies [21,22], while micro scale research is lacking from the perspective of specific agricultural products, but the eco-efficiency of rice production should be considered as this variable is an important part of the agricultural eco-efficiency. Second, regarding the methodology, although many researchers have performed environmental impact assessments of crop systems with the LCA method in China [8,9,10], there is a lack of research on measuring the eco-efficiency of rice production using the LCA and the DEA jointly. Moreover, a sensitivity analysis of LCA results should be considered to increase the credibility of the evaluation results, which has become a necessary step in LCA research. A sensitivity analysis focuses on how the selection and changes in data or methodology affect the results of the LCA. The uncertainty in the LCA refers to the uncertainty of results caused by model inaccuracy, uncertainty in inputs, and the accumulation of variability in data [23]. The sensitivity and uncertainty of evaluation results are lacking in many studies. In fact, the sensitivity analysis and uncertainty test of evaluation results should be conducted to enhance the credibility of the results. Based on the above research limitations, this paper evaluated the eco-efficiency of rice systems with both the LCA and DEA methods based on household survey data in Hubei Province in the middle reaches of the Yangtze River. Then, sensitivity analysis and scenario analysis were carried out on the comprehensive index of the rice environmental impact and eco-efficiency of rice systems, respectively. This article aims to scientifically evaluate and notably improve the eco-efficiency of rice systems to provide theoretical guidance and mitigate the environmental problems associated with single-cropping rice production systems in the middle reaches of the Yangtze River and improve sustainable rice development.

The rest of the paper is organized as follows. Section 2 presents the study area, the data and the approach, and it is followed by Section 3 that presents the empirical results. Section 4 presents the discussion, while Section 5 concludes with the innovation, limitations and future studies.

## 2. Materials and Methods

### 2.1. Study Area and Data

The sample area is located in the middle reaches of the Yangtze River, which belongs to the typical subtropical monsoon humid climate zone, with sufficient sunlight and abundant heat and precipitation. In this agricultural zone, rain and heat occur in the same season, with an annual average temperature of approximately 15–17 °C. The annual average precipitation ranges from 800–1600 mm, and precipitation is mainly concentrated in July and August [24]. Single-cropping rice is the current typical planting pattern in this region [25], and the field management measures are mainly characterized by high water consumption, high fertilizer application and intermittent irrigation. For a long time, the excessive input of pesticides and fertilizers in this region has not only greatly increased the cost of rice cultivation for farms but has also caused risk of notable environmental damage.

**Figure 1:**
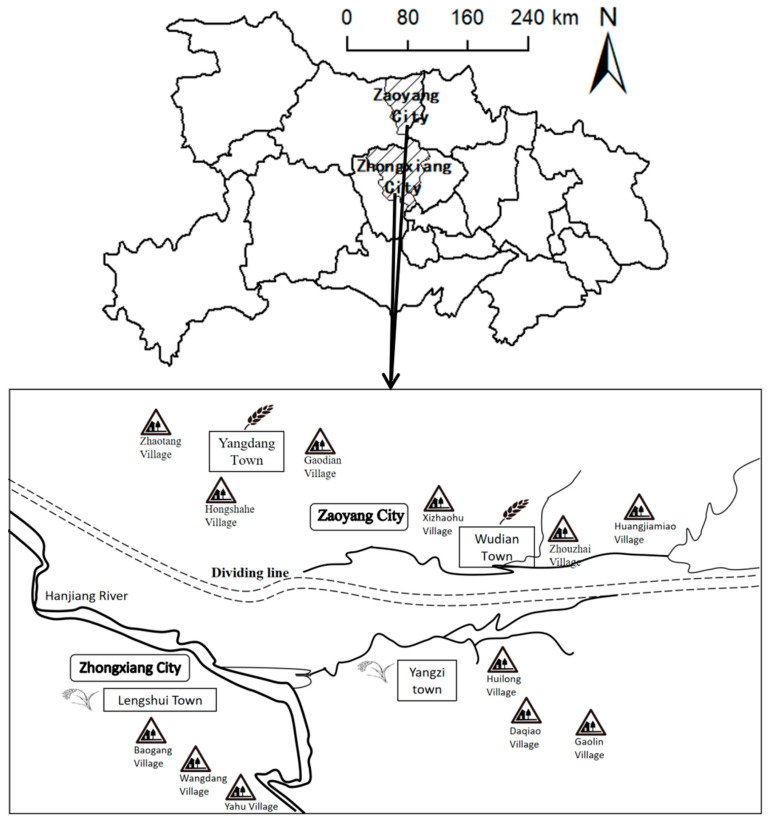
Sample area map.

In this study, we obtained data from a randomized rural household survey of Chinese smallholder farmsin December 2020, and their farms mainly grow rice. The information refers to the production year of 2020. Hubei is a major rice-growing province in China. In 2020, the sown area and production reached 2.29 thousand hectares and 18.77 million tons, accounting for about 7.70% and 8.96% of the sown area and production in the whole country, respectively, and both ranked fifth in the country [1]. The study sample sites, Zhongxiang City in the Jianghan Plain of southern Hubei and Zaoyang City in the down-land of northern Hubei, are both major regions of single-season rice systems in Hubei [26], which have good regional representation. Zhongxiang City is subordinate to Jingmen City, and Zaoyang City is subordinate to Xiangyang City. Jingmen City ranks 3rd in Hubei in terms of rice sown area and production in 2020. The rice sown area and production in Zhongxiang City is 70.4 thousand hectares and 0.65 million tons, accounting for 27.66% and 29.68% of the sown area and production in Jingmen City in 2020, respectively. Meanwhile, Xiangyang City ranks 5th in Hubei in terms of rice sown area and production in 2020. The rice sown area and production in Zaoyang City is 51.33 thousand hectares and 0.49 million tons, accounting for 25.42% and 26.29% of the sown area and production in Xiangyang City in 2020, respectively. The study considered the representative of the sample when choosing towns and villages. Specifically, the two selected towns are both large grain production towns, and the selected villages are also large grain production villages in the town; meanwhile their population, rice sown area and production are all ranked in the forefront. In addition, the study comprehensively considered time, humanpower and budget, then determined 2 towns, 3 villages in each town, and about 30–35 households in each village to conduct questionnaire surveys. Figure 1 showed the sample area map.

The survey adopts the combination of stratified sampling and random sampling, and the way of “one-on-one” interviews between investigators and respondents. Face-to-face interviews were conducted by well-trained census takers who spoke both Mandarin and local dialects using a detailed structured questionnaire. The census takers were all team members. The pre-test of the questionnaire was carried out before the formal investigation. The formal investigation is conducted according to the following procedures. At the first stage, two cities, Zaoyang and Zhongxiang, were selected based on their geographic characteristics and regional representation aspects. At the second stage, two towns within each selected city were randomly chosen, including Yangdang town and Wudian town in Zaoyang and Yangzi town and Leng shui town in Zhongxiang. At the third stage, three villages were randomly selected in each town. Finally, approximately 30–35 households were randomly selected and interviewed in each village. A total of 400 paper questionnaires were obtained. Finally, 30 questionnaires were considered invalid if they were unreturned, missing or if the participants stopped answering. These invalid questionnaires were excluded, and the total amount of valid questionnaires applicable to this study was 370 with an effective rate of 92.5%.

The survey gathered information covering household and farm-level characteristics (e.g., age, gender, education, farm size and household size), the use of production inputs (e.g., fertilizers, pesticides, plastic film, diesel and seeds), rice production and management, and green production patterns. We computed the sample size using Cochran’s sample size determination equation due to a lack of information on the smallholder rice household population in the sampled regions. This study refers to the studies of Zhou. et al. (2020) [27] and Ke and Shen (2015) [28], and uses Cochran’s sample size to determine the formula to calculate the minimum sample size. Cochran’s equation is expressed as N = Z^2^ × P × (1 − P)/E^2^, where we assume a margin of error e of 5%, a probability or *p* value of 0.5, and a confidence level of 90% with a corresponding Z value of 1.645, thus yielding a minimum sample size of N = 1.645^2^ × 0.5 × (1 − 0.5)/0.05^2^. This study relied on a sample size of 370 respondents to ensure precision. Basic information about the structure of these farms and the respondents, etc., are detailed in Table 1.

All material inputs and outputs of the rice production system are detailed in Table 2.

Here, the eco-efficiency of rice is measured, and evaluation index system construction is based on the relevant literature [29,30]. Considering the availability of data, the rice sown area, agricultural labor, total machinery cost, irrigation, fertilizers, pesticides and agricultural gasoline and diesel are selected as production input-related independent variables. The rice net output value is adopted as the expected output-related dependent variable. The comprehensive index of the environmental impact of the rice system assessed using the agricultural LCA method is applied as the unexpected output-related dependent variable. The final constructed eco-efficiency system of rice production is summarized in Table 3.

### 2.2. Agricultural Life Cycle Assessment

The International Society of Environmental Toxicology and Chemistry (SETAC) and the International Organization for Standardization (ISO) have defined the LCA as the compilation and evaluation of the input, output and potential environmental impacts throughout the life cycle of a given product system. The agricultural LCA method refers to the relationship between all material and energy inputs and outputs and measurable environmental loads due to agricultural production activities, and this approach aims to evaluate the total impact of resource consumption, energy consumption and agricultural production activities [31]. The evaluation includes four steps, namely, target definition and scope definition, inventory analysis, impact evaluation and result interpretation. In this paper, life cycle assessment of single-cropping rice systems in the middle reaches of the Yangtze River is chosen as the research objective, 1 ton of rice production is chosen as the evaluation functional unit, and the energy and material inputs, outputs and environmental impacts during the production life cycle of 1 ton of rice are analyzed. The starting boundary of the rice life cycle comprises the exploitation and transportation of raw materials and the production and transportation of agrochemicals related to the rice life cycle. The ending boundary encompasses the output of agricultural products and pollutants from sowing to harvesting during rice production.

In the inventory analysis process, resource consumption and the environmental emissions stemming from the exploitation of energy and raw materials are mainly considered at the stage involving raw and agricultural materials. Resource consumption and pollutant emissions originating from water and soil resources, fertilizers and pesticides are mainly considered at the farming stage. The technical indices of energy consumption and the emission coefficients of pollutants such as CO_2_, NH_3_, SO_2_, N_2_O and NO are mainly retrieved from Liang [31] and Wang [32]. Land utilization refers to the amount of land occupied for the production of 1 ton of rice. Water consumption only considers the total amount of irrigation consumption in farmlands. The effective components of N, P and K fertilizers were converted according to the effective components of compound fertilizers, ammonia bicarbonate and urea applied in the production process. The questionnaire recorded in detail the effective components of compound fertilizers N, P_2_O_5_ and K_2_O applied by farms during rice production and the effective components of N in ammonium bicarbonate and urea. The selection of N loss parameters in paddy fields, including NH3, NO_3_, NO_2_ and NO_X_, was based on Tian et al. [33], Yin et al. [34] and Brentrup et al. [35]. Regarding parameter selection for phosphorus loss determination in paddy fields, referring to Ji [36], the runoff loss rate of phosphorus nutrients is 0.86% of that of chemical fertilizers. Pesticide residue aspects were based on Calker et al. [37]. The pesticide residue pollutants entering the atmosphere, water and soil in this study accounted for 10%, 1% and 43%, respectively, of the input of effective components. The impacts of heavy metal pollution in farmlands on the environment only considered Cu, Zn, Pb and Cd introduced into farmlands by fertilizers and irrigation. Only the amount of heavy metals leaving the farming system in harvested grains was considered because all rice straw was returned to the paddy fields by the surveyed farms, similar to Ni [38]. The environmental impacts related to the system, such as plant equipment, building facilities, production of transportation tools and transportation of raw materials, were not considered.

The obtained comprehensive index of the environmental impact within the rice system can be incorporated into the eco-efficiency evaluation system of rice production as the unexpected output. Assessment of the ecological environmental impact via life cycle assessment consists of three steps: characterization, standardization and weighted assessment. First, equivalent coefficients, energy conversion coefficients, land use characterization parameters, etc., were employed in this paper to consider nine types [31,39] of environmental impacts: global warming, environmental acidification, eutrophication, human toxicity, water toxicity, soil toxicity, land use, energy consumption and freshwater consumption; an inventory of resource consumption and pollutant emissions was established. Next, we standardized the potential value of the environmental impact by considering the global per capita environmental impact potential in 2000 [40] as the benchmark value of the environmental impact. Then, the various environmental impact indices were summarized to obtain the comprehensive index of the environmental impact of the rice system based on weight coefficients determined in previous relevant research [39].

### 2.3. Unexpected Output Super Efficiency SBM Model

The eco-efficiency of rice production is the basic index to measure the level of rice ecological development and green production, which involves a functional trade-off among the inputs, outputs and ecological impacts. The eco-efficiency index of rice production includes expected and unexpected activities or outcomes.

This paper applies the gross output value of rice as the expected outcome and adopts the comprehensive index of the environmental impacts of the rice system during the life cycle as the undesired outcome. With DEA-SOLVER Pro 5.0 as the computing platform, the farms’ eco-efficiency of rice production is measured. Compared to the traditional DEA model, the SBM model can solve the problem of congestion or slack. However, the SBM model cannot distinguish efficiency differences between decision-making units (DMUs) with efficiency values equal to or exceeding 1. The super efficiency SBM model based on the unexpected output compensates for the abovementioned shortcomings [41]. Therefore, this paper applies the super efficiency DEA (SE-DEA) method to measure the eco-efficiency of rice production through the following model [42]:

(1) Min AEE=1m∑i=1m(x¯xik)1r1+r2(∑s=1r1yd¯/yskd+∑q=1r2yu¯/yqku)

(2) $$\left\{ \begin{array}{l} \bar{x}\geq \sum_{j=1,\neq k}^{n}x_{ij}\lambda_{j};\bar{y^{d}}\leq \sum_{j=1,\neq k}^{n}y_{sj}^{d}\lambda_{j};\bar{y^{d}}\geq \sum_{j=1,\neq k}^{n}y_{qj}^{d}\lambda_{j};\\ \bar{x}\geq x_{k};\bar{y^{d}}\leq y_{k}^{d};\bar{y^{u}}\geq y_{k}^{u}\\ \lambda_{j}\geq 0,i=1,2,\cdots ,m;j=1,2,\cdots ,n,j\neq 0;\\ s=1,2,\cdots ,r_{1};q=1,2,\cdots ,r_{2}; \end{array}\right.$$

Where *n* is the number of DMUs, namely the investigated farms, equal to 370. Each of which is composed of the inputs *m*, *r*_1_ is the expected output, *r*_2_ is the unexpected output, where *m* refers to the input of rice production factors, *r*_1_ is the net output value of rice, and *r*_2_ is the comprehensive index of the rice environmental impacts. *x*, *y^d^*, *y^u^*, are matrix elements of the input, expected output and unexpected output, respectively. *x* including land input, labor input, mechanical input, water input, fertilizer input, pesticide input and energy input.

## 3. Results

### 3.1. Comprehensive Index of the Rice Environmental Impacts

#### 3.1.1. Classification and Characterization

The variable impacts of the rice life cycle process can be divided into resource utilization and environmental impacts. Here, resource utilization mainly considers energy consumption, land utilization and water consumption, and the environmental impacts mainly include global warming, environmental acidification, eutrophication, human toxicity, water toxicity and soil toxicity. In this paper, the value of each environmental impact was obtained by calculating the potential ecological environmental impact throughout the rice life cycle (Table 4).

#### 3.1.2. Standardization and Weighted Evaluation

Water toxicity, soil toxicity, eutrophication and environmental acidification are the major environmental impacts throughout the rice life cycle, with the comprehensive index of the rice environmental impact of 12.57, 8.69, 1.78 and 0.28, respectively (Table 5). Notably, water toxicity, soil toxicity, eutrophication and environmental acidification attributed to the production of 1 ton of rice reached 1257%, 869%, 178% and 28%, respectively, which was equivalent to the world per capita environmental impact potential in 2000. The environmental impacts of rice energy consumption, human toxicity and water consumption are limited, all less than 4%, compared to the world per capita environmental impact potential in 2000. In the sample area, water toxicity, soil toxicity and eutrophication were the main influencing factors, accounting for 53.89%, 33.19% and 9.35% of the comprehensive index of the rice environmental impacts, respectively. The comprehensive index of the rice environmental impact within the rice life cycle is 2.0971. This value will be included as the unexpected outcome in the subsequent efficiency evaluation system of the super efficiency SBM model, which is considered to measure the farms’ eco-efficiency performance of rice production.

### 3.2. Eco-Efficiency of Rice Production

In this paper, farms are selected as the research unit. Battese [43] reported that a microscopic research object is more suitable for the variable return to scale (VRS) approach. Therefore, we choose the unexpected output super efficiency SBM-DEA model under VRS conditions to measure the eco-efficiency of rice production and apply the results under the limited VRS conditions as the analysis benchmark. The total weight of the expected output and the total weight of the unexpected output are set to 1. Details are listed in Table 6.

Different limiting conditions can lead to variable eco-efficiency values of rice production. In this paper, 370 decision-making units are calculated based on constant return to scale (CRS), general return to scale (GRS) and variable return to scale (VRS) considerations. The mean values of the eco-efficiency of rice production are 0.45, 0.48 and 0.51, respectively (refer to the last line of Table 6), when the total weight of the expected output and the total weight of the unexpected output are both set to 1. From CRS and GRS to VRS conditions, the limiting conditions are gradually relaxed in sequence, which is the main reason why the mean value of the eco-efficiency of rice production sequentially increases under the variable limiting conditions.

There are significant differences in the eco-efficiency of rice production among the various farms. This paper draws upon relevant research [44] and divides the eco-efficiency of rice production of the sampled farms into high-, medium- and low-eco-efficiency groups. More specifically, the high-efficiency group indicates that the high-efficiency state has been achieved, and farms production occurs at the forefront of production with an eco-efficiency of rice production equal to or higher than 1. Moreover, the eco-efficiency of rice production is lower than 1 if farms have not yet reached the production frontier, which belongs to the relatively ineffective DEA category. Therefore, there is a certain degree of efficiency loss. This paper further divides inefficient farms into the medium-efficiency group and low-efficiency group based on the distance of these farms to the production frontier. In particular, the eco-efficiency of rice production in the medium-efficiency group varied between 0.8 and 1, and that in the low-efficiency group was lower than 0.8. Based on the results in Table 6, it could be found that from CRS and GRS to VRS, with increasing relaxation of the limiting conditions, the number of farms in the low-efficiency group gradually decreased, while the number of farms in the medium and above (EE ≥ 0.8) efficiency group increased significantly. The reason for this result may be that the sample size of the study was limited. If this study expands the research area as well as the sample size to select more samples that fit the study, then a sample size from high eco-efficiency and medium eco-efficiency farms can be obtained. Thus, the sample will be more evenly distributed across different eco-efficiency groups and the empirical results will be more consistent with the theory. In this paper, the VRS model is still used as a benchmark for analysis, and the proportions of farms in the low-efficiency group, middle-efficiency group and high-efficiency group are 87.03%, 1.89% and 11.08%, respectively, with mean values of 0.42, 0.86 and 1.14, respectively. Therefore, there were two extreme phenomena in the eco-efficiency of rice production in the sampled area: the proportion of farms in the low-efficiency group was 6.7 times that in the middle- and high-efficiency groups. This suggests that there is a very high potential to expand the number of farms in the middle- and high-eco-efficiency groups and improve the regional eco-efficiency of rice production.

We further analyzed whether there were significant differences in fertilizer levels and pesticide inputs on the farms when the farms were in different groups of eco-efficiency performance. Therefore, we performed an ANOVA analysis.

According to the homogeneity of variance test, the significance levels of nitrogen fertilizer, P_2_O_5_, K_2_O and pesticide use were 0.015, 0.919, 0.435 and 0.040, respectively. Only nitrogen fertilizer and pesticide passed the 5% significance level test, with uneven variance. According to the results of the analysis of variance (Table 7), nitrogen fertilizer and pesticides passed the 5% significance level test, and there were statistically significant differences between groups, indicating that there were differences among the low-efficiency group, medium-efficiency group and high-efficiency group. That is, at least two groups have statistically significant effects on nitrogen fertilizer and pesticides.

Then, we learned more detailed information by performing a multiple comparison analysis. Because only nitrogen fertilizer and pesticide have significant differences between groups, and the variance is uneven, the article only shows the comparison results of Tamhane’s T2 method of nitrogen fertilizer and pesticide (Table 8).

In terms of nitrogen fertilizer use, there is a significant difference between the low eco-efficiency group and the high eco-efficiency group, with a significance of 0.000. In terms of pesticide use, there are significant differences between the low eco-efficiency group and the medium eco-efficiency group, the low eco-efficiency group and the high eco-efficiency group, with a significance of 0.048 and 0.001, respectively. Combined with the results of Appendix A Table A2, it can be concluded that the rice production of farms with medium eco-efficiency and high eco-efficiency has the more obvious characteristics of green production mode. Compared to the farms in the low eco-efficiency group, these farms have lower input levels of N, P_2_O_5_, K_2_O and pesticides. At the same time, the farms of the medium-efficiency group and the high-efficiency group have larger per unit yield, total yield level and sowing area.

### 3.3. Sensitivity Analysis

A sensitivity analysis of the LCA results is currently a necessary step in LCA research. Throughout the life cycle of rice systems, there are uncertainties in the number of raw materials consumed, diesel oil consumption and pollutant emissions, as well as pollutants discharged by farms at the application stage affected by rainfall and climate change. This paper verifies the impacts of changes in system input parameters on the ecological environmental impacts throughout the rice life cycle via sensitivity analysis, which is one of the main methodologies of uncertainty analysis. Specifically, we analyzed the sensitivity of chemical fertilizers and pesticides, which greatly contribute to the eco-efficiency of rice production. Assuming that the external inputs of chemical fertilizers and pesticides fluctuate by ±10%, ±20% and ±30%, while the other inputs remain unchanged, the impact of these changes in fertilizers and pesticides on the core indicators, such as the eco-efficiency of rice production, can be determined.

**Figure 2:**
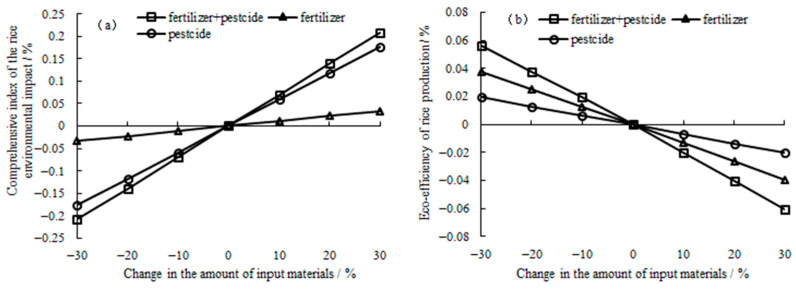
Sensitivity of the comprehensive environmental impacts (**a**) and eco-efficiency of the rice system (**b**).

A sensitivity analysis of the rice system environmental impact in Figure 2a demonstrates that the sensitivity of pesticides is higher than that of chemical fertilizers, and when fertilizers and pesticides simultaneously change, the sensitivity coefficient is the sum of the sensitivity coefficients due to the change in only the fertilizer or pesticide input. The sensitivity of pesticides is related to the toxicity to human health and ecological health caused by the excessive application of pesticides. The water toxicity and soil toxicity caused by the excessive application of pesticides are the highest, accounting for 53.89% and 33.19%, respectively, of the environmental impacts throughout the rice life cycle (Table 5). Thus, the sensitivity of pesticides is the highest. Excessive application of fertilizers causes the greatest harm to water eutrophication, but it only accounts for 9.35% of the environmental impacts throughout the rice life cycle (Table 5). In addition, the environmental impact on environmental acidification and energy consumption is relatively limited, thus the sensitivity of fertilizers is relatively low overall. A sensitivity analysis of the eco-efficiency of the rice production system indicates (Figure 2b) that after comprehensive consideration of the economic and environmental benefits of the rice production system, of the two inputs of fertilizers and pesticides, the sensitivity of chemical fertilizers is higher than that of pesticides. This mainly occurs because in the process of rice production, the amount of chemical fertilizers applied is much larger than that of pesticides, resulting in the input cost of fertilizers being much higher than that of pesticides, which leads to a corresponding lowering effect on the eco-efficiency of rice production that is far greater than that of pesticides. This also demonstrates that even though a certain type of agricultural chemical exerts little impact on crop systems, if this chemical is utilized excessively, it can also result in a great loss of the eco-efficiency.

### 3.4. Ways to Improve the Eco-Efficiency of Rice Production

The amount of fertilizers and pesticides used in major food crops in China far exceeds the optimal and economic application amounts needed [45,46,47]. In this study, three scenarios were set up to determine the potential for improving the eco-efficiency of the rice production systems in the sampled areas (Table 9). The eco-efficiency of rice production in 2020 is based on current fertilizer and pesticide management practices. Under Scenario 1, we reduced fertilizer consumption by 50% below the baseline. Due to the excessive application of fertilizers in the grain crop-planting process in China, fertilizer application optimization is the key point to improve eco-efficiency. Previous studies have reported that the excessive application of chemical fertilizers in grain production in China reaches 50% [48], and in the rice paddy areas in the middle and lower reaches of the Yangtze River, even more severe over-application of fertilizers occur [49]. Moreover, all the other inputs required for rice production remained the same as those used by farms in 2020. Under Scenario 2, it was assumed that the amount of pesticides used was reduced by 50% below the baseline. Although the actual utilization efficiency of pesticides among the three major food crops increased to 40.60% in China in 2020, the expected targets of pesticide reduction and utilization efficiency increase were successfully achieved. However, compared to developed countries, the actual utilization efficiency of pesticides in China remains very low. The European Commission issued the Farm to Table Strategy in 2020, which stated that the application of pesticides should be reduced by 50% in 2030 [50]. Therefore, there remains great potential to reduce pesticide application. Simultaneously, all the other inputs required for rice production remained the same as those under Scenario 1. Under Scenario 3, the determination of the eco-efficiency of rice production was based on the combination of pesticide reduction (Scenario 2) and fertilizer consumption optimization (Scenario 1).

This study reveals the great potential for improving the eco-efficiency of rice production. A scenario analysis demonstrates that the eco-efficiency of rice production reached 0.51 in 2020 (baseline). Compared to the baseline, the eco-efficiency of rice production could be increased by 4% to 0.53 by reducing the amount of fertilizers applied by 50% (Scenario 1). By reducing the amount of pesticides applied by 50% (Scenario 2), the eco-efficiency of rice production could be increased by 2%, up to 0.52. Moreover, the eco-efficiency of rice production could be increased by 6% to 0.54 when the application of both fertilizers and pesticides is reduced by 50%.

## 4. Discussion

### 4.1. Characteristics of the Eco-Efficiency of Rice Production

The overall eco-efficiency of rice production in the middle reaches of the Yangtze River is low. The average eco-efficiency value of rice production farms in this region was 0.51 in 2020. The eco-efficiency of rice production in the middle reaches of the Yangtze River is far lower than the average eco-efficiency of grain crops in China (0.81) [51], which is also lower than that of wheat (0.66) and corn (0.89) [52]. Moreover, the value is also lower than that of rice production in other countries (0.878–0.977) [53]. This further indicates that the eco-efficiency of rice production farms exhibits a considerable improvement potential in China. Based on previous studies, the varying performance of the eco-efficiency mainly depends on the system boundary, the amount of agricultural chemicals, the material input and the selected environmental impact factor [54,55]. Furthermore, some scholars analyzed the impact of fertilizer level and pesticide use on eco-efficiency, these findings all indicated that fertilizer level and pesticide use had a significant negative effect on agricultural eco-efficiency [56,57,58]. The present study further demonstrated that fertilizers and pesticides were the main contributors to the comprehensive index of the rice environmental impact. Water toxicity was mainly an environmental impact of pesticide loss at the farming stage. In addition to the environmental impact of pesticide loss at the farming stage, another important source of soil toxicity is the heavy metal pollution introduced into rice fields by fertilizer utilization and irrigation. Environmental acidification and water eutrophication were mainly caused by fertilizer production [16]. NH_3_ volatilization, NO_3_ leaching and P_2_O_5_ leaching and runoff [34] were also reasons for water eutrophication. Previous studies have reported that NO_3_ leaching [59] and N and P loss in fertilizer application [60] are important influencing factors of water eutrophication. Therefore, reducing the total input of fertilizers and pesticides is important to reduce environmental pollutant emissions and improve the eco-efficiency of rice production.

### 4.2. Improvement Potential of the Eco-Efficiency of Rice Production

Reducing agricultural nonpoint source pollution, lowering planting costs and improving the eco-efficiency and economic benefits of rice production can be effectively achieved by reducing fertilizer and pesticide inputs, improving the utilization efficiency and optimizing management measures. Although the government has been committed to guiding farms to carry out agricultural green production, agricultural nonpoint source pollution is still severe in China. Currently, many studies have reached a consensus that the application ratio of fertilizers and pesticides has exceeded the economically optimal application ratio in China. In 2016, the amount of fertilizers applied per unit of cultivated land in China reached 443.3 kg/hm^2^, which is 1.97 times the international safe application limit of fertilizers (225 kg/hm^2^). The pesticide utilization efficiency among the three major grain crops in China reached 40.6% in 2020, accounting for 50–60% of the ratio among wheat, corn and other types of grain crops in developed European countries and the United States. In this study, all farms applied ternary compound fertilizers, urea and ammonium bicarbonate, and the application ratio of organic fertilizers was 0%. The amounts of compound fertilizers, urea and ammonium bicarbonate per rice area reached 740.43 kg/hm^2^, 152.81 kg/hm^2^ and 121.40 kg/hm^2^, respectively. There were 203, 152 and 15 sampled farms spraying pesticides 3, 4 and 5 times, respectively, accounting for 54.86%, 41.08% and 4.06%, respectively, of all sampled farms. The average application amounts of pesticides were 9.75 kg/hm^2^, 14.1 kg/hm^2^ and 15.3 kg/hm^2^, respectively. Previous studies have demonstrated that farms exhibit a great potential to reduce fertilizers and pesticides in the middle and lower reaches of the Yangtze River. Excessive application of fertilizers by farms is severe, and the actual application amount of fertilizers is 1.43 times the optimal application ratio. Moreover, the degree of pesticide overuse is very severe, and the marginal pesticide productivity is close to zero [61].

Therefore, on the basis of reducing the production and application of fertilizers, especially nitrogen fertilizers, through optimization of fertilizer management, application of slow and controlled release fertilizer, addition of digestive inhibitors and application of organic fertilizers, the N_2_O emissions of nitrogen fertilizer application could be reduced. Moreover, the application of nitrogen fertilizers can be reduced, and greenhouse gas emissions in the fertilizer production process can also be lowered [62]. In terms of reducing pesticide application, we can decrease the application of highly and moderately toxic chemical pesticides and harmful additives, pesticides and water consumption, dependence on water sources and environmental pollution through comprehensive utilization of agronomic, physical, chemical, biological and ecological control measures [63]. Due to the typical characteristics of high-water consumption, high fertilizer application and flooding irrigation in the rice region of the middle reaches of the Yangtze River, optimization of the irrigation and fertilizer management modes [62] and planting modes [64] and adoption of scaled operations [65] cannot only directly improve the eco-efficiency of rice production but can also indirectly improve the eco-efficiency of rice production by reducing the input of agricultural chemicals. Therefore, agricultural production aims to ensure the effective supply of agriproducts and increase the income of farms, as well as reduce fertilizer and pesticide utilization and improve the utilization efficiency through the above measures, thereby simultaneously promoting the transition from an excessive dependence on agricultural resource consumption to pursue green production [66].

### 4.3. Uncertainty in the Eco-Efficiency of Rice Production

Compared to Figure 2a,b, when the amount of production materials was changed, the change in the comprehensive index of the environmental impact of rice system was larger than that in the eco-efficiency of rice production. The eco-efficiency of the rice production index can comprehensively reflect the dual goals of green production and food security. On the one hand, China is a country with a large population, and the goal of ensuring the absolute safety of domestic rations is firm and unwavering. On the other hand, because ecological security and environmental friendliness in food production are inevitable trends in modern agriculture, we cannot be biased. Rice environmental impact assessment can only evaluate the environmental load due to rice production but ignores economic benefits. The eco-efficiency of rice production can comprehensively assess the ecological and economic benefits of rice production, and it is also an important index to measure sustainable agriculture. Usually, rice production can reduce the environmental load by reducing the material input, but this may lower the economic benefits of rice production. Thus, based on reducing the utilization of fertilizers and pesticides, it is necessary to optimize irrigation and fertilizer management practices, pesticide chemistry and nonchemical control technology [67], large-scale planting [55] and other measures to improve the utilization efficiency of fertilizers and pesticides to achieve the dual goals of reducing the utilization of fertilizers and pesticides and improving the eco-efficiency.

Life cycle assessment (LCA) is an important tool to measure the eco-efficiency of products or organizations from a micro perspective. Agricultural systems also face the problem of sustainable development under dual resource and environmental pressures. Here, the theory and method of the agricultural LCA were introduced. In this study, a sensitivity analysis of the eco-efficiency of rice production revealed that compared to fertilizers, pesticides were more sensitive to the environmental impacts of rice systems, but fertilizers were more sensitive when considering the eco-efficiency of rice systems. This change was mainly caused by the uncertainty in various agricultural chemical inputs and emission factors. First, various data sources are key uncertainty factors in an environmental impact assessment and eco-efficiency analysis. The data analyzed in this paper were retrieved from household survey data and related literature, which could ensure regional differences and representativeness. However, there may also be measurement errors due to subjective bias and parameter inaccuracy aspects. In particular, certain key parameters are based on the calculation results of other countries, in which the production conditions and technologies are significantly different from those in China, resulting in a possible calculation deviation. Next, the wide regional heterogeneity in rice planting across China in terms of climate, geographical characteristics, cropping system, soil types and agricultural management practices may generate uncertainty in equivalence factors, and these factors and corresponding interaction effects make it difficult to formulate suggestions for national eco-efficiency improvement in China because our study only focused on single-cropping rice in the middle reaches of the Yangtze River. Thus, it is necessary to develop appropriate locally adapted accounting models according to regions, crop types and management practices in the future to generate calculation results that are closer to the real eco-efficiency level. The scenario analysis revealed the great improvement potential of the eco-efficiency of rice production systems. The eco-efficiency performance can be affected by the application technique and the timing of fertilizers and pesticides, as well as changes in the irrigation method and frequency [68]. Therefore, future studies should more deeply estimate the impact of these factors on the eco-efficiency of rice production systems. Nevertheless, this work still provides basic information on agricultural chemical inputs and their impact on environmental impact assessment and the eco-efficiency of rice production.

## 5. Conclusions and Policy Implications

The implementation of fertilizer and pesticide reduction and utilization efficiency increase is a key measure to realize the concept of sustainable development. Based on survey data pertaining to the Hubei Province in the middle reaches of the Yangtze River, this paper quantifies the relevant environmental impacts within the rice production boundary of farms and considers the main restrictive factors of agricultural production water and land resources using the agricultural LCA method. Then, the weighted comprehensive index of the environmental impacts was chosen as the unexpected output of the rice eco-efficiency evaluation system. On this basis, the super efficiency SBM model was employed to calculate the eco-efficiency of rice production. The paper draws the conclusions below, striving to provide empirical references to promote the reduction in fertilizers and pesticides and encourage overall green transformation of the agricultural production modes.

The environmental impacts throughout the rice life cycle mainly include water toxicity, soil toxicity, eutrophication and environmental acidification, and the comprehensive index of the rice environmental impact were 12.57, 8.69, 1.78 and 0.28, respectively. The comprehensive index of the environmental impacts throughout the rice life cycle was 2.0971 based on weighting assessment. Water toxicity, soil toxicity and eutrophication were the main contributing factors, accounting for 53.89%, 33.19% and 9.35%, respectively, of all environmental impacts. Water toxicity and soil toxicity were mainly caused by the extensive use of pesticides. Eutrophication and environmental acidification were mainly caused by the excessive application of fertilizers, especially nitrogen fertilizers.

Under the limiting conditions of the VRS model, and when the total weight of the expected output and the total weight of the undesired output were both set to 1, the eco-efficiency of rice production of farms reached 0.51. More specifically, the proportion of farms in the low-efficiency group, middle-efficiency group and high-efficiency group was 87.03%, 1.89% and 11.08%, respectively, with average values of 0.42, 0.86 and 1.14, respectively. The results revealed two extremes. Notably, the proportion of farms in the low-eco-efficiency groups was 6.7 times than that in the medium- and high-eco-efficiency groups. Therefore, we should focus on increasing the number of farms with a high eco-efficiency, promote the leading and exemplary role of farms with a high eco-efficiency of rice production, encourage farms with low and medium eco-efficiencies to continuously improve their eco-efficiency, and improve the overall eco-efficiency level of rice production regionally.

The eco-efficiency of rice production systems exhibits a great potential. Sensitivity analysis of the environmental impacts of rice production systems indicated that the sensitivity of pesticides was higher than that of fertilizers. However, after comprehensively weighing the environmental and economic benefits of rice production, it was found that the sensitivity of fertilizers was greater than that of pesticides. Meanwhile, scenario analysis showed simultaneous reducing both fertilizers and pesticides could notably improve the eco-efficiency of rice production. Thus, suggestions such as lowering the application of fertilizers and pesticides and improving the utilization efficiency of fertilizers and pesticides could be implemented to reduce environmental loads and improve the eco-efficiency.

There are some innovation aspects of this study. On one hand, at the research scale, rice production eco-efficiency is an important part of agro-ecological efficiency, but the present research on agricultural eco-efficiency in China is mainly focused on agricultural eco-efficiency in national and provincial level research due to the limitation of data acquisition. There is a lack of research on specific crops, eco-efficiency at the micro-scale using farms survey data. Therefore, as a case study, this study is beneficial to expand and enrich the study on agricultural eco-efficiency at the micro-scale. On the other hand, in terms of research methods, we jointly apply the life cycle assessment and DEA methods to measure rice eco-efficiency, then sensitivity analysis was conducted on the analysis results to enhancing the credibility of the results. Thus, this study is conducive to improving the research methods on agricultural eco-efficiency.

However, there are also some limitations in this study. Firstly, agriculture could collect and transform part of the carbon in its self-system operation and increase the carbon sink through photosynthesis, but this study only considers the environmental load of agricultural production, and does not consider its positive impact on the environment. Secondly, some parameters in the study were retrieved from the publications many years ago. The technical indices of energy consumption and the emission coefficients of pollutants were retrieved from publications in 2008 and 2009, as well as the world per capita environmental impact potential in 2000 was referred as the baseline value of environmental impact. Although the scope and geographical characteristics of these publications are similar to our research in terms of climatic conditions, natural conditions, and agricultural production conditions, etc., there are certain risks in timeliness and applicability when these coefficients are cited in this study more than 10 years later. Thirdly, China is a vast territory country, and rice is planted from south to north, but this study was analyzed only in Hubei Province in the middle reaches of the Yangtze River, which weakens the applicability of the findings and conclusions in the whole country.

For future studies, the study should make up for the above deficiencies if possible. Firstly, when calculating the environmental impact of crops, the carbon sink effect during the production of crops should be considered. Secondly, when using the agricultural life cycle assessment method for agricultural environmental impact evaluation, the study should try to use the data measured by field experiments, or quote the coefficients published in the latest publications. Thirdly, when measuring the agricultural eco-efficiency, it should start from a larger scale (nationwide), more watersheds, and more crop varieties as much as possible to get a more realistic eco-efficiency for the whole China

## Figures and Tables

**Table 1:** The samples’ basic statistical characteristics.

Variables	Definition	Mean	Std. Dev.	Min	Max
Gender	Gender of respondents: female = 0; male = 1	0.97	0.17	0	1
Age	Age of respondents (years)	57.88	8.67	31	79
Rice area	Rice sown area (hm^2^)	1.16	1.51	0.05	13.33
Per block area	The average area of each cultivated land (hm^2^)	0.13	0.09	0.01	1
Irrigation ratio	Proportion of paddy fields with irrigation condition (%)	82.97	16.44	50	100
Per labor capital	Average labor capital input of rice (yuan)	413.12	795.60	34.73	9179.57
Electric appliance	Quantity of electric appliances (PCs)	3.69	0.65	1	4
Agricultural machinery	Agricultural machinery is converted by following coefficients: cars = 1, rotary cultivators, rice trans planters, harvesters and walking tractors = 1, agricultural tricycles = 0.5, electric vehicles and motorcycles = 0.3, then summed up.	2.20	1.18	0	5.8
Market	Distance from home to the nearest market town (km)	4.07	2.37	0.2	15
House area	Residential area (m^2^)	252.55	246.96	40	1500
Labor capability	Labor capacity = whole labor × 1 + half labor × 0.5	2.60	1.02	0.5	6.5
Education	Average years of formal education for family labor (years)	8.53	2.17	0	13.5
Farming cooperative	Join the agricultural cooperative: no = 0, yes = 1	0.11	0.31	0	1
Rice disaster insurance	Purchase rice disaster insurance or not: no = 0, yes = 1	0.28	0.45	0	1
Per capita income	Annual per capita income (yuan)	24,274.99	21,123.41	1511.53	277,628.70
Credit	Access to credit or not: no = 0, yes = 1	0.18	0.38	0	1
Subsidy	Agricultural subsidy (yuan)	1975.02	1377.56	200	11,000

**Table 2:** Material input and production of rice in the sample area.

Inputs/ Outputs	Irrigation/ m^3^/hm^2^	N/ kg/hm^2^	P_2_O_5_/ kg/hm^2^	K_2_O/ kg/hm^2^	Pesticides/ kg/hm^2^	Seeds/ kg/hm^2^	Electricity/ kwh/hm^2^	Diesel Oil/ kg/hm^2^	Production/ kg/hm^2^
Mean	3592.68	241.06	87.34	111.60	11.79	31.47	742.08	54.81	10,375.60

**Table 3:** Evaluation index system of eco-efficiency of rice production.

Indicator Category	Classification Index	Variable Explanation	Remarks	Mean	SD
Factor of production	Land input	Rice planting area (hm^2^)	Reflects the actual planting area of rice production	1.158	1.506
	Labor input	Labor days of rice (man/man-day)	The total amount of labor employed in rice production is converted on a daily basis	55.774	62.053
	Mechanical input	Total cost of rice machinery services (yuan)	The cost of agricultural machinery services represents the level of mechanization utilization.	3234.669	4152.092
	Water input	Irrigation water consumption of rice (m^3^)	Total irrigation in rice production	4184.393	5673.659
	Fertilizer input	Fertilizer application amount (kg)	The fertilizer input is one of the main pollution sources in rice systems	1135.940	1553.249
	Pesticide input	Pesticide consumption (kg)	The pesticide input is one of the main pollution sources in rice systems	13.432	18.274
	Energy input	Usage of agricultural gasoline and diesel oil (kg)	Agricultural gasoline and diesel inputs are pollution sources in rice systems	47.308	75.963
Expected output	Rice output value	Net output value of rice (yuan) *	The total output value minus the total cost of rice planting in 2020	15,949.901	20,822.520
Unexpected output	Comprehensive index of the rice environmental impact	Environmental load caused by the rice life cycle process involving the input and consumption of N, P_2_O_5_, K_2_O, pesticides, agricultural electricity, agricultural gasoline and diesel, irrigation, land use, energy consumption and seeds	The comprehensive index of the rice environmental impact was estimated with the agricultural LCA method	22.996	24.572

* Note: The article has presented the original data for calculating the net output value of rice in Appendix A Table A1.

**Table 4:** Potential ecological environmental impacts throughout the rice life cycle.

Types of Ecological Environmental Impacts	Agricultural Resource System	Farming System	Value
Energy depletion/MJ	2530.662	1736.909	4267.571
Water consumption/m^3^	—	358.171	358.171
Land use/m^2^	—	997.440	997.440
Global warming/kg CO_2_-eq.	267.984	125.707	393.691
Acidification/kg SO_2_-eq.	1.708	12.919	14.627
$\mathrm{Eutrophication}/\mathrm{kg}\;{\mathrm{PO}}_{4}^{3-}$-eq	0.304	3.202	3.506
Human toxicity/kg 1,4-DCB-eq.	—	5.811	5.811
Water toxicity/kg 1,4-CDB-eq.	—	60.707	60.707
Soil toxicity/kg 1,4-CDB-eq.	—	53.122	53.122

Note: potential environmental impact from the production of 1 ton of rice.

**Table 5:** Standardized and weighted analysis of the potential environmental impacts throughout the rice life cycle.

Types of Environmental Impacts	Unit	Standardization Impact Index	Weighted Impact Index
Energy depletion	MJ/a	0.0016	0.0002
Water resource consumption	m^3^/a	0.0407	0.0045
Land resource utilization	m^2^/a	0.1839	0.0257
Global warming	kgCO_2_-eq	0.0573	0.0069
Environmental acidification	kgSO_2_-eq	0.2799	0.0336
Eutrophication	$\mathrm{kg}{\mathrm{PO}}_{4}^{3-}$-eq	1.7810	0.1959
Human toxicity	kg1,4-DCB-eq	0.0295	0.0035
Water toxicity	kg1,4-DCB-eq	12.5687	1.1312
Soil toxicity	kg1,4-DCB-eq	8.6943	0.6955
comprehensive index of the rice environmental impacts			2.0971

**Table 6:** Household eco-efficiency of rice production in the sampled area.

Group	CRS		GRS		VRS	
	Households	Mean Value	Households	Mean Value	Households	Mean Value
High-efficiency group (EE ≥ 1)	21	1.08	21	1.15	41	1.14
Medium-efficiency group (0.8 ≤ EE < 1)	6	0.84	19	0.90	7	0.86
Low-efficiency group (EE < 0.8)	343	0.40	330	0.42	322	0.42
Sample population	370	0.45	370	0.48	370	0.51

**Table 7:** Results of the ANOVA analysis.

		Sum of Squares	Degree of Freedom	Mean Square	F	Significance
N	Between-group	1044.55	2	522.27	8.18	0.0003
	Within-group	23,422.62	367	63.82		
	Total	24,467.17	369			
P_2_O_5_	Between-group	27.59	2	13.79	0.91	0.4033
	Within-group	5561.15	367	15.15		
	Total	5588.73	369			
K_2_O	Between-group	34.84	2	17.42	1.42	0.2434
	Within-group	4506.67	367	12.28		
	Total	4541.51	369			
Pesticide	Between-group	1.30	2	0.65	6.20	0.0023
	Within-group	38.34	367	0.10		
	Total	39.64	369			

**Table 8:** Multiple comparison results in different rice eco-efficiency groups.

Dependent Variable	(I) ID	(J) ID	Mean Difference (I–J)	Standard Error	Significance	95% Confidence Interval	
						Lower Limit	Upper Limit
N	Low-efficiency group	Medium-efficiency group	0.8848	3.5635	0.993	−10.6468	12.4165
		High-efficiency group	5.3578 *	0.9177	0.000	3.1132	7.6024
	Medium-efficiency group	Low-efficiency group	−0.8848	3.5635	0.993	−12.4165	10.6468
		High-efficiency group	4.4730	3.6218	0.593	−7.0035	15.9495
	High-efficiency group	Low-efficiency group	−5.3578 *	0.9177	0.000	−7.6024	−3.1132
		Medium-efficiency group	−4.4730	3.6218	0.593	−15.9495	7.0035
pesticide	Low-efficiency group	Medium-efficiency group	0.2115 *	0.0674	0.048	0.0017	0.4214
		High-efficiency group	0.1692 *	0.0446	0.001	0.0596	0.2787
	Medium-efficiency group	Low-efficiency group	−0.2115 *	0.0674	0.048	−0.4214	−0.0017
		High-efficiency group	−0.0424	0.0765	0.931	−0.2561	0.1714
	High-efficiency group	Low-efficiency group	−0.1692 *	0.0446	0.001	−0.2787	−0.0596
		Medium-efficiency group	0.0424	0.0765	0.931	−0.1714	0.2561

* The significance level of the mean value difference is 0.05.

**Table 9:** Improvement potential of the eco-efficiency via rice production scenario analysis.

	Fertilizer Change Ratio	Pesticide Change Ratio	Eco-Efficiency of Rice Production Value	Eco-Efficiency of Rice Production Change Ratio
original value	-	-	0.5117	-
Scenario 1	−50%	-	0.5319	3.94%
Scenario 2	-	−50%	0.522	2.01%
Scenario 3	−50%	−50%	0.5414	5.79%

## Data Availability

The data will be made available to the reader upon request.

## Appendix A

**Table A1:** Rice cost and revenue data for sample farms.

Variables	Unit	Mean	Std. Dev.	Min	Max
Gross income	yuan	27,996.95	35,981.48	1404.00	330,000.00
Irrigation cost	yuan	913.21	1957.38	40.00	20,000.00
Compound fertilizer cost	yuan	2369.89	3080.81	96.00	30,000.00
Nitrogen fertilizer cost	yuan	98.81	358.69	0.00	4130.00
Urea cost	yuan	331.07	595.53	0.00	6000.00
Pesticides cost	yuan	1641.04	2166.38	80.00	17,280.00
Seed cost	yuan	2657.95	3458.42	0.00	28,000.00
Electricity cost	yuan	363.47	520.46	18.96	4680.00
Labor cost	yuan	436.95	1524.04	0.00	18,200.00
Tillage and land preparation cost	yuan	1409.23	1969.34	72.00	20,000.00
Seeding cost	yuan	308.97	851.25	0.00	12,000.00
Harvesting cost	yuan	1516.47	2043.44	0.00	20,000.00
Total cost of rice	yuan	12,047.04	16,103.65	554.16	150,000.00
Net profit	yuan	15,949.90	20,822.52	849.84	170,000.00
Yield	ton	11.91	15.17	0.60	130.00
Net income per ton	yuan	1311.63	277.91	318.17	1962.38

**Table A2:** Descriptive statistical analysis of different rice eco-efficiency groups.

		N	Mean	S.D.	S.E.	Min	Max
production (ton)	Low-efficiency group	322	9.459	8.698	0.485	1.000	88.500
	Medium-efficiency group	7	23.691	16.375	6.189	2.700	42.000
	High-efficiency group	41	29.157	33.143	5.176	0.600	130.000
	Total	370	11.911	15.171	0.789	0.600	130.000
sown area (hm^2^)	Low-efficiency group	322	0.929	0.829	0.046	0.107	7.867
	Medium-efficiency group	7	2.159	1.591	0.601	0.200	4.000
	High-efficiency group	41	2.783	3.423	0.535	0.053	13.333
	Total	370	1.158	1.506	0.078	0.053	13.333
yield (ton/hm^2^)	Low-efficiency group	322	10.215	1.770	0.099	5.250	15.000
	Medium-efficiency group	7	11.821	1.427	0.539	9.998	13.500
	High-efficiency group	41	11.309	1.996	0.312	6.000	15.750
	Total	370	10.366	1.830	0.095	5.250	15.750
N (kg/t)	Low-efficiency group	322	24.550	8.253	0.460	10.714	67.179
	Medium-efficiency group	7	23.665	9.349	3.534	14.353	37.683
	High-efficiency group	41	19.192	5.085	0.794	5.442	29.639
	Total	370	23.939	8.143	0.423	5.442	67.179
P_2_O_5_ (kg/t)	Low-efficiency group	322	8.840	3.908	0.218	3.214	30.000
	Medium-efficiency group	7	7.435	3.265	1.234	4.706	14.066
	High-efficiency group	41	8.189	3.854	0.602	2.268	22.500
	Total	370	8.741	3.892	0.202	2.268	30.000
K_2_O (kg/t)	Low-efficiency group	322	11.120	3.570	0.199	2.000	30.000
	Medium-efficiency group	7	9.780	2.493	0.942	7.529	14.066
	High-efficiency group	41	10.305	3.073	0.480	3.628	22.500
	Total	370	11.004	3.508	0.182	2.000	30.000
Pesticide (kg/t)	Low-efficiency group	322	1.197	0.332	0.019	0.650	2.686
	Medium-efficiency group	7	0.985	0.172	0.065	0.722	1.253
	High-efficiency group	41	1.028	0.260	0.041	0.684	1.625
	Total	370	1.174	0.328	0.017	0.650	2.686
